# Emerging pneumococcal carriage serotypes in a high-risk population receiving universal 7-valent pneumococcal conjugate vaccine and 23-valent polysaccharide vaccine since 2001

**DOI:** 10.1186/1471-2334-9-121

**Published:** 2009-08-04

**Authors:** Amanda J Leach, Peter S Morris, Gabrielle B McCallum, Cate A Wilson, Liz Stubbs, Jemima Beissbarth, Susan Jacups, Kim Hare, Heidi C Smith-Vaughan

**Affiliations:** 1 Menzies School of Health Research, John Mathews Building (Bldg58), Royal Darwin Hospital, Rocklands Drive, Tiwi, Northern Territory, Australia; 2 Charles Darwin University, Ellengowan Drive, Casuarina, Northern Territory, Australia; 3 NT Clinical School, Royal Darwin Hospital, Rocklands Drive, Tiwi, Northern Territory, Australia

## Abstract

**Background:**

In Australia in June 2001, a unique pneumococcal vaccine schedule commenced for Indigenous infants; seven-valent pneumococcal conjugate vaccine (7PCV) given at 2, 4, and 6 months of age and 23-valent pneumococcal polysaccharide vaccine (23PPV) at 18 months of age. This study presents carriage serotypes following this schedule.

**Methods:**

We conducted cross sectional surveys of pneumococcal carriage in Aboriginal children 0 to 6 years of age living in remote Aboriginal communities (RACs) in 2003 and 2005. Nasal secretions were collected and processed according to published methods.

**Results:**

902 children (mean age 25 months) living in 29 communities in 2003 and 818 children (mean age 35 months) in 17 communities in 2005 were enrolled. 87% children in 2003 and 96% in 2005 had received two or more doses of 7PCV. From 2003 to 2005, pneumococcal carriage was reduced from 82% to 76% and reductions were apparent in all age groups; 7PCV-type carriage was reduced from 11% to 8%, and 23PPV-non-7PCV-type carriage from 31% to 25% respectively. Thus non-23PPV-type carriage increased from 57% to 67%. All these changes were statistically significant, as were changes for some specific serotypes. Shifts could not be attributed to vaccination alone. The top 10 of 40 serotypes identified were (in descending order) 16F, 19A, 11A, 6C, 23B, 19F, 6A, 35B, 6B, 10A and 35B. Carriage of penicillin non-susceptible (MIC > = 0.12 μg/mL) strains (15% overall) was detected in serotypes (descending order) 19A, 19F, 6B, 16F, 11A, 9V, 23B, and in 4 additional serotypes. Carriage of azithromycin resistant (MIC > = 2 μg/mL) strains (5% overall), was detected in serotypes (descending order) 23B, 17F, 9N, 6B, 6A, 11A, 23F, and in 10 additional serotypes including 6C.

**Conclusion:**

Pneumococcal carriage remains high (~80%) in this vaccinated population. Uptake of both pneumococcal vaccines increased, and carriage was reduced between 2003 and 2005. Predominant serotypes in combined years were 16F, 19A, 11A, 6C and 23B. Antimicrobial non-susceptibility was detected in these and 17 additional serotypes. Shifts in serotype-specific carriage suggest a need more research to clarify the association between pneumococcal vaccination and carriage at the serotype level.

## Background

Australian Aboriginal children in remote communities experience high rates of many infectious diseases[1,2], particularly pneumococcal disease.[3] Concern about antibiotic resistance is used as an argument against greater use of antibiotics. Recent reports describe escalating rates of disease caused by pneumococcal serotypes not included in currently licensed pneumococcal conjugate vaccine (7PCV),[3,4]including an increasing proportion of isolates having antibiotic resistance. [5-7] Serotype 19A is of particular interest in Australia[3] and internationally.[8] More recent data suggests 6C as a new and emerging replacement serotype, and that 7PCV is cross-protective for 6A. Prior to 7PCV introduction in 2001,[9] pneumococcal carriage in Indigenous infants was around 80% and approximately 60% were 7PCV serotypes (unpublished data from 3 of the communities also in these surveys). In 2003 and 2005 we conducted wider geographic surveillance of pneumococcal carriage.

## Methods

### Vaccine schedule

From July 2001 7PCV was recommended and funded for Australian Aboriginal and other high-risk infants at 2, 4 and 6 months of age. In the Northern Territory and in South Australia, 23-valent pneumococcal polysaccharide vaccine (23PPV) was recommended as a booster dose for Aboriginal children at 18 months of age.[9] Children less than 2 years of age were offered catch-up 7PCV.

### Study setting and participant eligibility criteria

Participating communities were in four regions of South Australia and the Northern Territory. Children were eligible if they were Aboriginal, 0 to 6 years of age, resident in a participating community, and if their parents or caregiver provided written consent.

### Swab collection, antibiotic use and vaccination history

Nasal secretions were collected using swabs or nose blowing into a tissue as previously described.[10,11] Pneumococcal vaccination dates and antibiotics prescribed in the 5 weeks prior to swab collection were obtained from the NT childhood immunization register and participant medical records.

### Microbiological analysis

Nasal secretions were transported, stored and processed as previously described.[10] Pneumococci were identified and one or more colonies (if morphologically distinct) selected for serotyping with antisera from the Statens Serum Institute (Denmark). Antimicrobial susceptibility was determined by disc diffusion[12] and Etest (AB Biodisk, Sweden). We performed PCR for the *wciN *gene.[13]

### Statistical analysis

Confidence intervals (CI, 95%) and risk differences (RD, with 95% CI) were calculated where appropriate. Stata version 10 was used for all data analysis[14]

### Ethical Approval and Funding

The study was approved by the regional Human Research Ethics Committees and Aboriginal Sub-Committee with right of veto of the Northern Territory Department of Health and Community Services and Menzies School of Health Research. The study was funded by the National Health and Medical Research Council.

## Results

### Participant characteristics

Nasal secretions were collected from 902 children in 29 communities in 2003 and from 818 children in 17 communities in 2005 (Table 1). Nasal swabs comprised 94% specimens. Mean age was 30 months. 83 were less than 6 months of age.

**Table 1:** Participant characteristics

	2003	2005	Total
**No. Children**	902	818	1720
**Mean age in months (95% CI)**	25 (24–26)	35 (33–36)	30.0 (29–31)
**Sex male (%)**	447 (50%)	403 (49%)	850 (49%)

### Vaccine uptake

In 2003 87% children had received at least 2 doses of 7PCV (Table 2). In 2005, this figure was 96% (Risk Difference = 9% [95% Confidence Interval 7,12]). The proportion of children receiving a full primary 7PCV plus booster 23PPV course increased from 35% to 62% (RD = 27% [22,31]) (Tables 2 and 3). 169 children had no record of receiving either 7PCV or 23PPV. Ten children had 23PPV only. Of the 83 infants less than 6 months of age, 59 (71%) had received at least one dose of 7PCV.

**Table 2:** Vaccination status, pneumococcal carriage by serotype, recent antibiotic use and carriage of non-susceptible pneumococci, by year

Year	2003	2005		
No. communities	29	17		
No. children swabbed	902	818		
	Children (%)		Risk Difference	P
Vaccinated (>1 dose)	87%	96%	9% [7,12]	0.0000
Vaccinated (primary)	76%	84%	9% [5,12]	0.0000
Vaccinated (primary+23v)	35%	62%	27% [22,31]	0.0000
**Spn (% children)**	82%	76%	-6% [-10,-2]	0.002
**0–6 mo n = 83**	83%	74%	-9.7 [-28,8]	0.3098
**6–12 mo n = 220**	80%	78%	-1.6 [-13,10]	0.7791
**12–24 mo n = 477**	84%	76%	-8.2 [-16, -0.2]	0.0331
**24–48 mo n = 597**	84%	78%	-5.3 [-12,1]	0.0989
**48- n = 343**	77%	74%	-2.9 [-13,7]	0.5677
**7PCV-type**	11%	8%	-4% [-6, -0.1]	0.0105
				
**23PPV-type**	42%	33%	-9% [-14,-5]	0.0001
**23PPV-type, non-7PCV**	31%	25%	-6% [-10,-2]	0.0061
**19A**	12%	9%	-3% [-6,-1]	0.0196
**Non-23PPV**	57%	67%	9% [5,14]	0.0001
**6A**	4%	2%	-1.5% [-3,0.1]	0.0712
**6C**	4%	4%	0% [-2,2]	0.9634
**16F**	12%	10%	-2% [-5,1]	0.1343

**Prescribed beta-lactam**	14%	16%	1% [-2,5]	0.4763
**Penicillin non-susceptible (MIC ≥ 0.12 μg/mL)**	17%	14%	-3% [-6,0.4]	0.0899
**Prescribed macrolide**	1%	6%	5% [3,7]	0.0000
**Azithromycin resistant (MIC ≥ 2 μg/mL)**	5%	6%	1% [-1,3]	0.4888
**Azithromycin resistant (MIC ≥ 32 μg/mL)**	4%	3%	-1% [-3,1]	0.2328

**Table 3:** Pneumococcal (Spn) carriage, 7-valent pneumococcal conjugate vaccine (7PCV) uptake, carriage of 7PCV and 23PPV types, recent antimicrobial use, and carriage of non-susceptible pneumococci in children 0 to 6 years of age, by region and year.

Region	Central Australia		Darwin Rural^¥^		East Arnhem		Katherine West	
Year	2003	2005	2003	2005	2003	2005	2003	2005
No. communities	11	2	6	6	7	4	5	5
No. children swabbed	158	65	320	376	315	223	109	154
	Percent children (95% Confidence Interval)							

**Spn positive**	83(77–89)	86(78–95)	79(75–84)	67(62–72)*	85(81–89)	87(83–92)	82(74–89)	78(71–85)

**Vaccinated (> = 1 dose)**	96(93–99)	97(93–101)	88(84–91)	96(94–98)*	80(76–84)	97(95–99)*	89(83–95)	94(90–98)

**Vaccinated (primary)**	85(79–90)	83(74–92)	79(75–84)	87(84–90)*	71(66–76)	82(77–87)*	70(61–78)	82(76–89)

**Vaccinated (primary+23v)**	46(39–54)	54(41–66)	34(29–39)	65(60–69)*	30(24–34)	59(52–65)*	39(29–48)	62(54–69)*

**7PCV-type positive**	10(5–14)	12(4–21)	15(11–19)	9(6–12)	10(7–13)	10(6–14)	6(2–11)	3(0–5)

**Serotype in 23PPV not in 7PCV**	41(33–49)	40(29–52)	33(27–38)	25(21–29)	26(21–33)	23(17–28)	30(22–39)	31(23–38)

**19A**	14(8–19)	15(6–24)	17(12–21)	10(7–13)	8(5–11)	6(3–9)	5(1–9)	10(5–14)

**6A**	0.6(-1–2)	3(-1–7)	3(1–5)	2(0–3)	6(4–9)	4(2–7)	6(1–10)	1(0–3)

**6C**	3(0–6)	2(-2–5)	4(2–7)	3(1–5)	6(3–8)	7(3–10)	2(-1–4)	5(2–9)

**16F**	10(5–15)	11(3–19)	12(8–15)	12(8–15)	11(7–14)	10(6–14)	19(12–27)	6(2–9)*

**Prescribed beta-lactam**	28(21–35)	38(26–51)	17(13–21)	16(12–20)	7(4–9)	12(7–16)	9(4–15)	10(5–14)

**Penicillin non-susceptible (MIC ≥ 0.12 μg/mL)**	25(19–32)	42(29–54)	18(14–22)	9(6–12)*	14(10–18)	16(11–21)	13(6–19)	13(8–18)

**Prescribed macrolide**	0	2(-2–5)	3(1–5)	13(9–16)*	0	0	2(-1–4)	2(0–4)

**Azithromycin resistant (MIC ≥ 2 μg/mL)**	9(4–12)	22(11–32)	3(1–5)	5(3–8)	3(1–4)	3(1–5)	11(5–17)	5(1–8)

*95% confidence interval not overlapping with previous year. MIC = Minimum inhibitory concentration.

^¥^Note: One large community had participated in a trachoma eradication program involving mass azithromycin treatment in the weeks before our 2005 survey; carriage was 48% (22/45) in children with a record of recent treatment and 51% (36/70) in children with no record of recent treatment.

### Carriage

Pneumococcal carriage in 2003 and 2005 combined was 79%. There was no difference in pneumococcal recovery between nasal swab (79%) and nose blowing (80%) collection methods. Pneumococcal carriage declined overall from 82% in 2003 to 76% in 2005 (RD = -6% [-10, -2] p = 0.0037), and similar reductions were seen in all age groups (Table 2). However this was not seen in all regions (Table 3).

#### Carriage in children seen in both surveys

Of the 226 children seen in both surveys, 80% were positive each year, and 148 (65%) were carriage positive in both 2003 and 2005; only 11 carried the same serotype.

#### Carriage in communities seen only in 2003

In the 12 communities seen only in 2003, 106 (82%) of 130 children were carriage positive, 5% carried azithromycin resistant strains (MIC > = 2 μg/mL) and 23% carried penicillin non-susceptible strains (MIC > = 0.12 μg/mL). Carriage and resistance in the other 17 communities seen in 2003 was 82%, 5% and 17%, respectively.

#### Serotype-specific carriage

**Figure 1:**
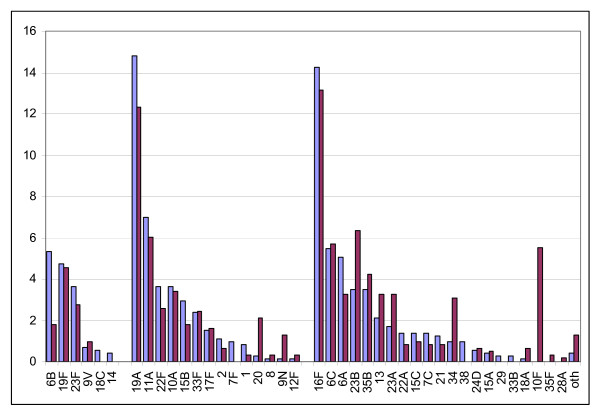
**Proportion of children with nasopharyngeal carriage of pneumococcal serotypes, by 7PCV and 23PPV serotype group and year**. Significant reductions in 6B and 19A from 2003 to 2005. Significant increases in 20,9N,23B, 34,10F from 2003 to 2005.

7PCV-type carriage was 11% in 2003 and 8% in 2005 (RD = -4% [-6, -0.1] p = 0.0105) (Table 2). 23PPV -non-7PCV-type carriage was 42% in 2003 and 33% in 2005 (RD = -6% [-10,-2] p = 0.0061]). Reductions reached statistical significance for serotypes 6B and 19A. Non-23PPV-type carriage increased overall (RD = 9% [CI 5,14] p = 0.0001) and reached statistically significance for non-vaccine types 23B, 23A, 34 and 10F, and for 23PPV types 20 and 9N. Of the isolates serotyped, the predominant carriage serotypes were 16F(14%), 19A(13%), and 11A (6.5%) and 6C (6%) (see Figure 1).

#### Non-vaccinated children

In 2003 and 2005, carriage in 169 non-vaccinated children (no 7PCV or 23PPV) was 74% and 70% respectively; carriage of 7PCV types was 9% and 7%, 23PPV-non-7PCV types was 19% and 24%. Predominant serotypes were 16F(10%) and 19A(9%) followed by 6% each of 11A, 6C, 23B and 22F.

#### Infants less than 6 months of age

Carriage in 83 infants less than 6 months of age was 83% in 2003 and 74% in 2005 (RD = -10% [-28, 8]). Carriage of 7PCV types was 20% and 4%, respectively (RD = -16% [-31, -1]). Predominant serotypes were 19A(17%), 16F(14%), 6A(8%) and 11A(8%) followed by 6B, 15C and 22F.

#### Non-vaccinated infants less than 6 months of age

For 24 non-vaccinated infants less than 6 months of age (mean age 1.9 months), carriage was 43% (3/7) and 65% (11/17) in 2003 and 2005, respectively (RD = 22% [-21, 65]). The 2003 serotypes were 6B, 11A and 20. The 2005 serotypes were 6A and 6C(2) and one each of 10F, 11A, 13, 18A, 19A, 19F, 33F, 34, and 22F.

#### 23PPV vaccinated children

Although carriage of 23PPV-non-7PCV types declined from 2003 to 2005, this could not be explained by the rise in 23PPV uptake. For both years combined, among children more than 18 months of age, carriage of 23PPV-non-7PCV types was 30% in 23PPV-vaccinated children compared to 26% in non-vaccinated children (RD = 4% [-2,10]). This difference was statistically significant in 2003 (RD = 11% [3,19]), but not in 2005 (RD = -1% [-12,9]). At the serotype level, these trends were present for serotype 19A but did not reach statistical significance.

### Antibiotic use in the 5 weeks prior to swab collection

Beta-lactam antibiotics had been recently prescribed (within 5 weeks prior to swabbing) for 14% children in 2003 and for 16% in 2005 (Tables 2 and 3). In general, macrolides were rarely recently prescribed with the exception of 48 of 396 (13%) children the Darwin Rural region (Table 3). Of these 48 children, 45 were from one community and had been prescribed azithromycin for trachoma eradication.

### Antibiotic resistance

Carriage of penicillin non-susceptible pneumococci (MIC ≥ 0.12 μg/mL) was 17% in 2003 and 14% in 2005 (Table 2). In Central Australia where prescribing was highest, carriage was 25% in 2003 and 42% in 2005 (Table 3). Penicillin resistance of 1.0 μg/mL or more was found in six children from 3 communities, all were serotype 9 V. Azithromycin resistant pneumococci (MIC ≥ 2 μg/mL) were carried by 5% children in 2003 and 6% in 2005. In the community where mass azithromycin had been recently used, 6 of 45 (13%) children with a record of recently prescribed azithromycin carried an azithromycin-resistant strain compared to 5 of 70 (7%) children with no record of recently prescribed azithromycin (RD = 6% [-5, 18]).

### Antibiotic-resistant serotypes

**Figure 2:**
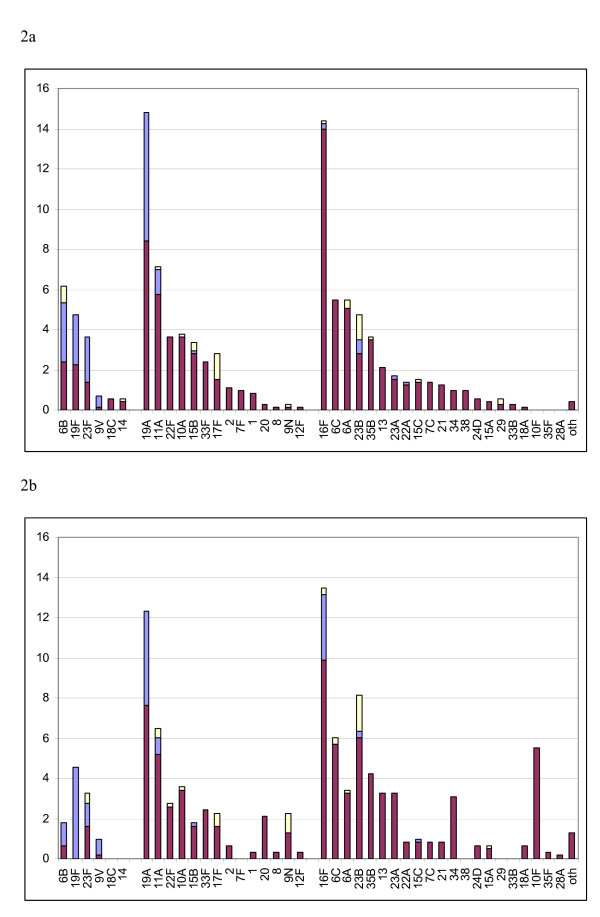
**Proportion of children with nasopharyngeal carriage of pneumococcal serotypes and antimicrobial non-susceptible serotypes, 7PCV and 23PPV serotype group**. A: 2003. B: 2005. Serogroups: Left – 7PCV serotypes. Middle – 23PPV serotypes. Right – non-23PPV serotypes. MacR (yellow): azithromycin MIC ≥ 2 μg/mL. PenR (mauve): penicillin MIC ≥ 0.12 μg/mL. Strains with resistance to both beta-lactam and macrolide antibiotics were included in each category. (in 2003 six of 38 6B isolates, two of 25 23Bs and one of 50 11As; in 2005 one of 39 23B isolates and one of 37 11As.)

Penicillin resistance (MIC> = 0.12 μg/mL) was found in both 7PCV and non-7PCV serotypes (Figure 2). In 2003, 34% of 137 resistant isolates were serotype 19A and in 2005, 26% (of 112 isolates) were 19A. Of 24 resistant isolates in Central Australia in 2005 9 (38%) were serotype 19A. Serotype 16F accounted for 7% in 2003 and 19% in 2005.

Macrolide resistance (azithromycin MIC ≥ 2 μg/mL) was found mainly in the non-7PCV serotypes (Figure 2). Eight isolates with MIC > = 256 μg/mL were found in 6 communities in regions 1 and 2 in 2003. Serotypes were 16F(2) and 6B(6).

## Discussion

Our survey of 1720 young children in remote communities two and four years after commencement of a government funded schedule for Indigenous children of 7PCV at 2, 4 and 6 months followed by a booster 23PPV at 18 months of age, provides a valuable source of pneumococcal serotype and susceptibility data for the region. Two major influences have had the potential to impact on pneumococcal carriage in this population; pneumococcal vaccination and antimicrobial prescribing. In three communities studied extensively in the 1990s, around 50% children carried 7PCV serotypes. Predominant pre-7PCV serotypes were 6B, 19F, 23F, 19A, 16F and 6A (unpublished data from 3 of the communities also in these surveys). Retrospective PCR analysis confirms one of 20 pre-7PCV 6A isolates was 6C.[15] A similar serotype distribution was found in cultures of discharge from acute perforations of the tympanic membrane.[16]

In general, this survey found small differences between 2003 and 2005. Vaccine uptake (at least 2 doses of 7PCV) was around 87% in 2003 and increased to 96% in 2005. By 2005, 84% age-eligible children had received 23PPV. Between 2003 and 2005 we detected small but statistically significant reductions in pneumococcal carriage (-6%). This trend was consistent across all age groups. Similarly for 7PCV types (-4%). There was a small reduction in 6A. Overall, carriage of 23PPV-non-7PCV types was significantly reduced (-5%), including serotype 19A (-4%), but other 23PPV-non-7PCV types (20 and 9N) increased. Non-vaccine serotypes (not in 23PPV) increased by 10%, mainly attributable to serotypes 23B, 34 and 10F. Serotypes 6C and 16F remained unchanged.

Among the group of children not vaccinated, 19A, 16F and 6A and 6C were the predominant serotypes and 7PCV type carriage was less than 10%, indicating a herd effect. Similarly, in very young non-vaccinated infants (mean age 1.9 months) carriage was around 58% and only 8% carried 7PCV vaccine types. Serotypes were diverse and no dominant emerging serotype was evident in this young group.

There is no strong evidence from 23PPV studies to suggest that 23PPV has an impact on NP carriage[17] Our immunogenicity study[18] showed 23PPV to be an effective booster for 7PCV-primed serotypes, and that modest responses to some additional serotypes in 23PPV also occurred. Whether the antibody concentrations achieved would prevent pneumococcal acquisition in the nasopharynx was not assessed. In this survey we did detect a 5% reduction in 23PPV-non-7PCV serotype carriage between 2003 and 2005, at a time when uptake of 23PPV as a booster dose increased from 35% to 62%. In the age group eligible for 23PPV booster, carriage of serotypes unique to 23PPV was actually higher in 23PPV-vaccinated than non-vaccinated children, but only in 2003. We are unable to explain this observation which was not present in 2005. Whether this is related to lack of effect of 23PPV on carriage, or timing of the swabbing in relation to 23PPV vaccination, or other factors cannot be assessed from this study. Further studies of 23PPV seem warranted as is large-scale surveillance of 23PPV-non-7PCV serotypes in this population.

Beta-lactam prescribing did not change significantly between 2003 and 2005, however the region with highest prescribing also had higher carriage of penicillin non-susceptible strains. Serotypes 19A and 16F accounted for a considerable proportion of beta-lactam- non-susceptible strains, as did remaining 7PCV serotypes. The finding that penicillin non-susceptibility is linked to the major endemic replacement serotypes indicates a need for ongoing monitoring to assess trends over coming years. High level penicillin resistance was very uncommon in 2005 (1 isolate). The clinical impact of penicillin resistance will be much greater if high level resistance increases over time. In the 2008 report by Roche[3], serotype 19A caused the majority (36%) of penicillin non-susceptible IPD cases in Australia in 2006.

Macrolide prescribing was low, but increased significantly between 2003 and 2005, mainly due to recent mass azithromycin use in one community. At the time of our survey in that community there was no statistically significant difference in pneumococcal carriage or carriage of azithromycin resistant strains between children recently prescribed azithromycin and those not (RD = 6% [-5, 18]). Our previous longitudinal study[19] had shown that azithromycin reduces carriage of pneumococci, providing resistant strains with a selective advantage that allows spread within the community. Over time, and in the absence of further azithromycin use or selective pressure, susceptible stains re-populate and the proportion of the population carrying resistant strains declines.[19] The present cross-sectional study does not capture this dynamic process and therefore does not allow any conclusions regarding the relationship between mass azithromycin use and carriage. In Central Australia, recent macrolide prescribing was rare, but carriage of azithromycin resistant strains was clearly higher than other regions. We suggest that this higher background of resistance may be related to a longer period of exposure to azithromycin for trachoma[20] and STI (sexually transmitted infection)[21] eradication programs, in the central Australian region. Data to support this are lacking; the 2006 Trachoma surveillance report identified that azithromycin treatment was recommended but was poorly documented in the Northern Territory[20]

Whilst carriage prevalence does not fully predict IPD serotypes (particularly outbreak or epidemic serotypes such as serotypes 1 or 5), the majority of annual IPD cases are caused by the serotypes most prevalent in the carriage population. In Australia, rates of IPD caused by serotype 19A in Indigenous children have fluctuated over recent years and serotype 6A became the most common non-vaccine IPD serotype in Indigenous children and the second most common in non-Indigenous children.[3] Hanna[22] compared IPD serotypes in under 5 year old Indigenous children living in far North Queensland pre- and post-7PCV implementation. They concluded that there was no evidence of non-7PCV serotype replacement. However, the pre-vaccine non-7PCV cases were inflated by a serotype 1 outbreak. Excluding type 1 cases, there was a risk difference in non-7PCV IPD of 55% [95%CI 30,80]. Reporting of pneumococcal epidemiology needs to consider the epidemic and endemic nature of various serotypes and that serotype-specific data are needed to best describe and prepare for shifts over time and in response to vaccines and antibiotic selective pressures.

Otitis media (OM) serotypes closely align with carriage serotype profile,[23] and for Aboriginal children OM is a common and serious infection often resulting in chronic suppurative OM (CSOM). This study showed that the serotypes colonising infants at 2 months of age, and therefore the serotypes most likely causing early OM,[24] are no longer 7PCV-types. Vaccines that are effective in preventing early onset of ear disease (and pneumonia) are urgently needed.

## Conclusion

Pneumococcal disease epidemiology is in a transition period in Australia. This surveillance shows that nasal carriage of 7PCV serotypes has been replaced by the serotypes that were the next most common carriage serotypes prior to 7PCV use – namely 19A and 16F. Antibiotic resistance is almost exclusively restricted to these serotypes (and remaining 7PCV serotypes) and requires monitoring at the regional level. The role of 23PPV in limiting carriage and preventing disease in children also needs further investigation. More work is needed to guide design of appropriate surveillance systems that measure serotype shifts and distinguish serotype replacement from secular trends.

## Competing interests

The authors declare that they have no competing interests.

## Authors' contributions

AL, PM & HSV conceived of the study, participated in its design, securing funding and coordination and helped to draft the manuscript. GM and CW managed participant recruitment and specimen collection. LS coordinated laboratory studies. KH conducted bacteriology and pneumococcal serotyping. JB assisted with the bacteriology and database management. SJ assisted with the statistical analysis. All authors read and approved the final manuscript.

## Pre-publication history

The pre-publication history for this paper can be accessed here:

http://www.biomedcentral.com/1471-2334/9/121/prepub

## References

[B1] Rothstein J Heazlewood R Fraser M Health of Aboriginal and Torres Strait Islander children in remote Far North Queensland: findings of the Paediatric Outreach Service Med J Aust 2007 186 519 521 17516899 10.5694/j.1326-5377.2007.tb01026.x

[B2] Clucas DB Carville KS Connors C Currie BJ Carapetis JR Andrews RM Disease burden and health-care clinic attendances for young children in remote aboriginal communities of northern Australia Bull World Health Organ 2008 86 275 281 10.2471/BLT.07.043034 18438516 PMC2647416

[B3] Roche P Krause V Cook H Invasive pneumococcal disease in Australia, 2006 Communicable Diseases Intelligence 2008 32 1 18 30 18522302 10.33321/cdi.2008.32.3

[B4] Singleton RJ Hennessy TW Bulkow LR Hammitt LL Zulz T Hurlburt DA Invasive pneumococcal disease caused by nonvaccine serotypes among alaska native children with high levels of 7-valent pneumococcal conjugate vaccine coverage JAMA 2007 297 1784 1792 10.1001/jama.297.16.1784 17456820

[B5] Farrell DJP Klugman KPM Pichichero MM Increased Antimicrobial Resistance Among Nonvaccine Serotypes of Streptococcus pneumoniae in the Pediatric Population After the Introduction of 7-Valent Pneumococcal Vaccine in the United States. [Article] Pediatric Infectious Disease Journal 2007 26 123 128 10.1097/01.inf.0000253059.84602.c3 17259873

[B6] Hanage WP Huang SS Lipsitch M Bishop CJ Godoy D Pelton SI Diversity and Antibiotic Resistance among Nonvaccine Serotypes of Streptococcus pneumoniae Carriage Isolates in the Post-Heptavalent Conjugate Vaccine Era J Infect Dis 2007 195 347 352 10.1086/510249 17205472

[B7] Pai R Moore MR Pilishvili T Gertz RE Whitney CG Beall B Postvaccine genetic structure of Streptococcus pneumoniae serotype 19A from children in the United States Infect Immun 2005 192 1988 1995 10.1086/498043 16267772

[B8] Moore MR Gertz RE Jr Woodbury RL Barkocy-Gallagher GA Schaffner W Lexau C Population snapshot of emergent Streptococcus pneumoniae serotype 19A in the United States J Infect Dis 2008 197 1016 1027 10.1086/528996 18419539

[B9] Australian Immunisation Handbook8th Edition 2003 2007 220 234

[B10] Leach AJ Stubbs E Hare K Beissbarth J Morris PS Comparison of nasal swabs with nose blowing for community-based pneumococcal surveillance in healthy children J Clin Microbiol 2008 46 2081 2082 10.1128/JCM.00048-08 18385438 PMC2446850

[B11] Stubbs E Hare K Wilson C Morris P Leach AJ Streptococcus pneumoniae and noncapsular Haemophilus influenzae nasal carriage and hand contamination in children: a comparison of two populations at risk of otitis media Pediatr Infect Dis J 2005 24 423 428 10.1097/01.inf.0000160945.87356.ca 15876941

[B12] Bell SM The CDS disc method of antibiotic sensitivity testing (calibrated dichotomous sensitivity test) Pathology 1975 7 Suppl-48 772573 10.3109/00313027509082602

[B13] Park IH Pritchard DG Cartee R Brandao A Brandileone MC Nahm MH Discovery of a new capsular serotype (6C) within serogroup 6 of Streptococcus pneumoniae J Clin Microbiol 2007 45 1225 1233 10.1128/JCM.02199-06 17267625 PMC1865839

[B14] StataCorp 2008 Stata Statistical Software: Release 10.0. College Station, Texas. Stata Corporation

[B15] Hare KM Smith-Vaughan HC Binks M Park IH Nahm MH Leach AJ 'Dodgy 6As' Differentiating pneumococcal serotype 6C from 6A using the Quellung reaction J Clin Microbiol 2009 47 6 1981 1982 19357203 10.1128/JCM.00046-09 PMC2691095

[B16] Leach AJ Microbiology of acute otitis media with perforation in Indigenous children Streptococci – New insights into an Old Enemy Proceedings of the XVIth Lancefield International Symposium on Streptococci and Streptococcal Diseases Ed Kadaba S Sriprakash 2006 89 92

[B17] Moberley S Mulholland EK Leach AJ Morris PS Carapetis JR Balloch A Maternal and infant antibody following pneumococcal vaccination during pregnancy

[B18] Leach AJ Morris PS Mackenzie G McDonnell J Balloch A Carapetis J Immunogenicity for 16 serotypes of a unique schedule of pneumococcal vaccines in a high-risk population Vaccine 2008 26 3885 3891 10.1016/j.vaccine.2008.05.012 18562052

[B19] Leach AJ Shelby-James TM Mayo M Gratten M Laming AC Currie BJ A prospective study of the impact of community-based azithromycin treatment of trachoma on carriage and resistance of Streptococcus pneumoniae Clin Infect Dis 1997 24 356 362 9114185 10.1093/clinids/24.3.356

[B20] Tellis B Keeffe JE Taylor HR Surveillance report for active trachoma, 2006: National Trachoma Surveillance and Reporting Unit Commun Dis Intell 2007 31 366 374 18268876 10.33321/cdi.2007.31.38

[B21] Bowden FJ Fethers K "Let's not talk about sex": reconsidering the public health approach to sexually transmissible infections in remote Indigenous populations in Australia Med J Aust 2008 188 182 184 18241182 10.5694/j.1326-5377.2008.tb01569.x

[B22] Hanna J Humphreys JL Murphy DM Invasive pneumococcal disease in Indigenous people in north Queensland: an update, 2005–2007 MJA 2008 189 43 46 18601643 10.5694/j.1326-5377.2008.tb01897.x

[B23] Leach AJ Morris PS The burden and outcome of respiratory tract infection in Australian and Aboriginal children Pediatr Infect Dis J 2007 26 S4 S7 10.1097/INF.0b013e318154b238 18049380

[B24] Leach AJ Boswell JB Asche V Nienhuys TG Mathews JD Bacterial colonization of the nasopharynx predicts very early onset and persistence of otitis media in Australian aboriginal infants Pediatr Infect Dis J 1994 13 983 989 7845752 10.1097/00006454-199411000-00009

